# Data on litter quality of host grass plants with and without fungal endophytes

**DOI:** 10.1016/j.dib.2016.04.030

**Published:** 2016-04-21

**Authors:** P.E. Gundel, M. Helander, L.A. Garibaldi, B.R. Vázquez-de-Aldana, I. Zabalgogeazcoa, K. Saikkonen

**Affiliations:** a IFEVA - CONICET – Faculty of Agronomy, Buenos Aires University (UBA), Argentina; b Section of Ecology, Department of Biology, University of Turku, 20100 Turku, Finland; c Natural Resources and Biomass Production Research, Natural Resources Institute Finland (Luke), 20520 Turku, Finland; d Grupo de Investigación en Agroecología (AGRECO), Sede Andina, Universidad Nacional de Río Negro (UNRN) and Consejo Nacional de Investigaciones Científicas y Técnicas (CONICET), Mitre 630, Río Negro, CP 8400 San Carlos de Bariloche, Argentina; e Department of Abiotic Stress, Instituto de Recursos Naturales y Agrobiología de Salamanca (IRNASA), Consejo Superior de Investigaciones Científicas (CSIC), Salamanca, Spain

## Abstract

Certain Pooideae species form persistent symbiosis with fungal endophytes of *Epichloë* genus. Although endophytes are known to impact the ecology and evolution of host species, their effects on parameters related with quality of plant biomass has been elusive. This article provides information about parameters related with the quality of plant litter biomass of two important grass species (*Schedonorus phoenix* and *Schedonorus pratensis*) affected by the symbiosis with fungal endophytes (*Epichloë coenophiala* and *Epichloë uncinata*, respectively). Four population origins of *S. phoenix* and one of *S. pratensis* were included. Mineral, biochemical and structural parameters were obtained from three samples per factors combination [species (and population origin)×endophyte]. This data can be potentially used in other studies which, by means of ‘data reanalyzing’ or meta-analysis, attempt to find generalizations about endophyte effects on host plant litter biomass. The present data is associated with the research article “Role of foliar fungal endophytes on litter decomposition among species and population origins” (Gundel et al., In preparation) [1].

**Specifications Table**

**Table:** 

Subject area	*Biology*
More specific subject area	*Plant-microbe interaction*
Type of data	*Tables and figures*
How data was acquired	*Minerals: ICP-OES (inductively coupled plasma optical emission spectrometry) method. C and N: dry combustion (Dumas) method by Leco TruMac CN- analyzer, Leco Corporation, USA. ADF and ADL: Ankom Automated Fiber Analyzer A2000. Alkaloids: HPLC.*
Data format	*Raw and filtered*
Experimental factors	*Plant species and origin, and symbiosis with fugal endophyte*
Experimental features	*Three plant tissue samples per combination of experimental factors were analyzed for mineral, biochemical and structural characterization.*
Data source location	*Ruissalo Botanical Garden, University of Turku, Finland*
Data accessibility	Data are presented in this article.

**Value of the data**

• The data present detailed information about effects of fungal endophytes on parameters related with litter biomass quality in two host grass species (two cultivars and three wild populations).
• Mineral, biochemical, and structural characteristics of biomass quality determine, among other ecological processes, litter decomposition in nature.
• This detailed information can be reused in future works looking for general patterns of fungal endophyte effects on host biomass quality and litter decomposition.

## 1 Data

Raw data of mineral, biochemical (alkaloids) and structural characterization of biomass litter produced by two plant species and populations [*Schedonorus pratensis*: the cultivar ‘Kasper’ (from Finland); and *Schedonorus phoenix*: the cultivar Kentucky-31 (from U.S.) and three wild origin (Gotland, Åland and Södermanland)] with (E+) and without (E−) fungal endophytes are presented in the included excel file online appendix. The file contains three sheets. The sheet ‘Chemistry’ contains the results of all analyzed minerals (Ca, Cu, Fe, K, Mg, Mn, P, S, and Zn) and structural parameters (Dry matter, Ash, ADF and ADL) in the three processed samples per population [i.e. each combination of species (population) and endophyte]. The next sheet ‘N and C’, presents results from three samples per population of percentage of nitrogen and carbon for each population. Finally, the sheet ‘Alkaloids’ contains results of alkaloid concentration (i.e. peramine and ergovaline) in E+ population (two analyzed samples per population) and a control analysis to confirm that E− populations were free of alkaloids. In this paper, Appendix A, Appendix A show mean values of each parameter (Fig. S1: K, S, P, Mn, Mg, Ca, Cu, Fe and Zn; and Fig. S2: Dry matter, ash, ADF and ADL) for each population (Appendix A, Appendix A). Data of nitrogen, carbon, and C:N ratio are presented in the associated research article [1].

## 2 Experimental design, materials and methods

### 2.1 Plant material and experimental design

The plant material used for analyzing the quality of biomass as affected by fungal endophytes was produced by plants growing in a common garden at Ruissalo Botanical Garden (University of Turku, Finland). Besides the commercial cultivar ‘Kentucky-31’, seeds of *S. phoenix* (tall fescue) were collected from three geographic locations around the Baltic Sea: Åland, (Finland), Gotland and Södermanland (Sweden). From each seed lot (i.e. population), a part of the collected seeds was treated with heated water in order to kill the fungus and to obtain endophyte-free plants. Thus, endophyte-symbiotic (E+; untreated) and non-symbiotic seeds (E−; treated) were obtained for each population. Ten individual plants of each population and symbiotic status were placed at random in a grid with 1 m^2^ for each plant in 2005 (for details see: [2], [3]). Following a similar design, 10 individual plants of *S. pratensis* (meadow fescue; cultivar ‘Kasper’), symbiotic (E+) and non-symbiotic (E−) with endophyte, were planted in 2008 (for details see: [4]). Along the successive years, all plants were checked under light microscope for endophyte presence or absence to confirm the nominal symbiotic status.

At the end of the growing season (autumn 2011), the aboveground biomass of plants from each combination of species, population, and symbiotic status were harvested. The biomass belonging to the 10 plants from each treatment was pooled and mixed. Three samples per treatment containing air-dried leaves and pseudostems were taken to run the analyses for biomass characterization.

### 2.2 Biomass characterization

Samples were analyzed in terms of carbon content and mineral composition (Ca, Cu, Fe, Mg, Mn, N, K, P, S and Zn). Mineral composition was determined by the ICP-OES (inductively coupled plasma optical emission spectrometry) method. Total carbon and nitrogen were determined with an automated dry combustion method (Dumas method) by Leco TruMac CN- analyzer, Leco Corporation, USA. (Details are included in the associated research article [1]). Acid detergent fiber [ADF: cellulose+lignin+ash (minerals and silica)] and acid detergent lignin (ADL: lignin) were determined by using the filter bag technique, with an Ankom Automated Fiber Analyzer A2000, based on the analytical method by Goering and Van Soest [5].

Fungal alkaloid concentration was determined on one sample of biomass per treatment. Ergovaline and its isomer ergovalinine were quantified by HPLC following a modification of the methods described by Hill et al. [6] and Yue et al. [7]. Peramine alkaloid concentration was determined using the HPLC method described by Barker et al. [8] and Yue et al. [9] (see [1]).

## Footnotes

Appendix A Supplementary data associated with this article can be found in the online version at doi:10.1016/j.dib.2016.04.030.
