# Supplementary material for: Data on litter quality of host grass plants with and without fungal endophytes
Source: Data Brief. 2016 Apr 21;7:1469–72. doi: 10.1016/j.dib.2016.04.030 (PMC4857214; doi:10.1016/j.dib.2016.04.030)
Supplement: Supplementary file 1 — Supplementary material [file mmc1.docx]

April 7, 2016

Buenos Aires, Argentina

To whom it may concern

Data in Brief

‘Conflicts of interest: none’

Sincerely yours,

Pedro E. Gundel
