# Supplementary material for: Data on litter quality of host grass plants with and without fungal endophytes
Source: Data Brief. 2016 Apr 21;7:1469–72. doi: 10.1016/j.dib.2016.04.030 (PMC4857214; doi:10.1016/j.dib.2016.04.030)
Supplement: Supplementary file 3 — Supplementary material [file mmc3.docx]

Figure S1. Concentration of mineral elements in symbiotic (E+: dark bars) and non-symbiotic (E-: white bars) plant litter from *Schedonorus pratensis* (cultivar ‘Kasper’) and *Schedonorus phoenix* (cultivar ‘Kentucky-31’ and wild origins Åland, Gotland and Södermanland). Values are mean ± S.E. (n = 3).

Figure S2. Dry weight of biomass, ash, ADF (acid detergent fiber) and ADL (acid detergent lignin) in symbiotic (E+: dark bars) and non-symbiotic (E-: white bars) litter from *Schedonorus pratensis* (cultivar ‘Kasper’) and *Schedonorus phoenix* (cultivar Kentucky-31, Åland, Gotland and Södermanland). Values are mean ± S.E. (n=3).

Table S1 shows individual analysis of variance testing the effect of each factor [plant species (and population origin) by endophyte symbiotic status] on each parameter (K, S, P, Mn, Mg, Ca, Cu, Fe, Zn, Dry matter, ash, ADF and ADL) characterizing the quality of plant biomass (Table S1).

Table S1. Analysis of variance evaluating the effect of origin and endophyte symbiosis on biomass quality parameters [mineral elements (Ca, Cu, Fe, K, Mg, Mn, P, S and Zn); and dry matter, ash, ADF (acid detergent fiber) and ADL (acid detergent lignin)] of *Schedonorus pratensis* (cultivar ‘Kasper’) and *Schedonorus phoenix* (cultivar ‘Kentucky-31’, and three wild origins Åland, Gotland and Södermanland plants).

|  | ***df*** | **F value** | **Pr(>F)*** | **F value** | **Pr(>F)*** | **F value** | **Pr(>F)*** | **F value** | **Pr(>F)*** |
| --- | --- | --- | --- | --- | --- | --- | --- | --- | --- |
|  |  | Ca | | Cu | | Fe | | K | |
| Origin | 4 | 285.3 | *** | 56.7 | *** | 60.6 | *** | 606.7 | ******* |
| Endophyte | 1 | 0.4 |  | 1.2 |  | 83.9 | *** | 106.2 | ******* |
| Ori x End | 4 | 98.9 | *** | 50.7 | *** | 26.8 | *** | 56.1 | ******* |
|  |  | Mg | | Mn | | P | | S | |
| Origin | 4 | 94.9 | *** | 1388.8 | *** | 117.2 | *** | 298.8 | ******* |
| Endophyte | 1 | 69.9 | *** | 192.0 | *** | 2.8 | . | 92.6 | ******* |
| Ori x End | 4 | 40.0 | *** | 57.5 | *** | 39.5 | *** | 211.9 | ******* |
|  |  | Zn | | Dry matter | | Ash | | ADF | |
| Origin | 4 | 26.9 | *** | 4.6 | ** | 112.6 | *** | 13.5 | ******* |
| Endophyte | 1 | 0.9 | . | 0.3 | . | 139. | *** | 0.0 | **.** |
| Ori x End | 4 | 0.6 | . | 1.2 | . | 56.8 | *** | 3.4 | ***** |
|  |  | ADL | |  | |  | |  | |
| Origin | 4 | 4.9 | ** |  |  |  |  |  |  |
| Endophyte | 1 | 0.0 | . |  |  |  |  |  |  |
| Ori x End | 4 | 4.7 | ** |  |  |  |  |  |  |
| ***** Codes: ‘***’ 0.001; ‘**’ 0.01; ‘*’ 0.05; ‘.’ 0.1; ‘ ’ 1 | | | | | | | | | |
